# A Practice to Be Corrected

**Published:** 1859-11

**Authors:** 


### "A PRACTICE TO BE CORRECTED."

The first article of the August number of the "*American Dental Review*" is headed as above. We agree mainly with the writer, but just now refer to a different practice. For instance, Dr. Leslie, of the Review, obstinately persists in writing our name "Watts,"

although he is aware that he once procured for us a slight castigation by so doing. So persistently obstinate is he, that when the "head lines" of the "*Review*" should read, "*Fang Filling.—Broch*," he has them, "*Fang Filling.—Watts*." Now, if he don't quit it, we'll——write a favorable "notice" of *Leslie's* Dental Depot. W.

